# Impact of Economic Shocks on Cumulative Advantage Processes: Recession, Recovery, and Trajectories of Inequality

**DOI:** 10.1093/geroni/igab046.1957

**Published:** 2021-12-17

**Authors:** Stephen Crystal

**Affiliations:** Rutgers University, Princeton, New Jersey, United States

## Abstract

This study compares the effect of the 2008 recession and subsequent recovery across generational cohorts by evaluating age-cohort trajectories of income inequality. Using data from the 2007 to 2016 waves of the Survey of Consumer Finances, we examine the trajectory of inequality for the overall population and by cohort in years spanning the Great Recession and subsequent recovery. We find that increases in per-capita income and wealth observed at the population-level during the recovery were not reflected among households below the median, leading to increasing inequality. Within cohorts, we observe growing inequality within cohorts in their primary working years. Findings are consistent with a model of integrative cumulative dis/ advantage, which predicts increasing within-cohort inequality over the life course influenced both by persistent micro- and macro-level processes of increasing heterogeneity. Our analyses highlight the potential role of extreme business cycle fluctuations, booms and busts, to exacerbate this underlying process.

